# Recovery from sport-induced muscle damage in relation to match-intervals in major events

**DOI:** 10.3389/fspor.2024.1422986

**Published:** 2024-07-17

**Authors:** Kazunori Nosaka, Trevor C. Chen

**Affiliations:** ^1^ School of Medical and Health Sciences, Centre for Human Performance, Edith Cowan University, Joondalup, WA, Australia; ^2^ Department of Physical Education and Sport Sciences, National Taiwan Normal University, Taipei, Taiwan

**Keywords:** World Cup, Olympic Games, grand slam, delayed onset muscle soreness, muscle function, performance, injury

## Abstract

Muscle damage could affect the next match performance in sports when the time to recover from a previous match is shorter. We examined the interval between matches in nine team sports (e.g., soccer, rugby, field hockey, basketball, volleyball, baseball) and two racket sports (badminton, tennis) in World Cups held in 2022-2023, 2020 Tokyo Olympic Games and Gland Slam in 2023. We then performed narrative review using three electronic databases (PubMed, Scopus, Google Scholar) to get information about muscle damage and recovery in the 11 sports, and discussed whether the intervals in the events would be enough for athletes. We found that the match intervals varied among sports and events ranging from 0 to 17 days. The interval was the shortest for softball (0–2 days) and the longest (5–17 days) for rugby. Regarding muscle damage, changes in muscle function and/or performance measures after a match were not reported for cricket, volleyball and softball, but some information was available for other sports, although the studies did not necessarily use athletes who participated in the major events. It was found that recovery was longer for soccer and rugby than other sports. Importantly, the match-intervals in the events did not appear to accommodate the recovery time required from the previous match in many sports. This could increase a risk of injury and affect players’ conditions and health. Changing the match-intervals may be difficult, since it affects the budget of sporting events, but an adequate interval between matches should be considered for each sport from the player’s and coach's point of view.

## 1 Introduction

Major sporting events such as World Cups, Olympic Games, and Gland Slam in tennis attract millions of people worldwide (1). In these events, athletes perform multiple matches in a certain time period, especially when they progress to quarterfinal, semifinal and final matches. However, it is not necessarily clear how matches are scheduled in the sporting events. Limited information is available for the recovery time in relation to the match schedule.

Movements consisting of eccentric (lengthening) contractions (i.e., eccentric-biased movements) of leg and other muscles are performed in sports, which could induce muscle damage representing by delayed onset muscle soreness (DOMS) and prolonged decreases in muscle functions, even for well-trained athletes (2–4). Muscle damage impairs performance for several days, affecting subsequent training sessions and next matches (5–9). For example, Chou et al. (3) reported that it took 4–5 days for maximal voluntary isometric contraction (MVIC) strength of the knee extensors and flexors and performance measures such as Yo-Yo intermittent recovery test level one to return to baseline following a simulated soccer (football) match using a 90 min Loughborough Intermittent Shuttle Test (LIST) performed by elite female soccer players. Since it is likely that official soccer matches are more demanding than the LIST, it is assumed that soccer players require at least 4 days between matches to be ready for the next match (8). However, the interval between soccer matches does not necessarily appear to be longer than 4 days in major events such as World Cups and Olympic Games.

The time taken for recovery from a match varies among sports (3, 5, 10–26). For example, Souglis et al. (5) compared soccer, basketball, volleyball and handball at an elite competitive level for muscle damage and inflammatory indices. They showed that soccer produced the greatest increases in inflammatory markers, creatine kinase (CK) and lactate dehydrogenase (LDH) activities in the blood, and these were smallest after volleyball. Abián et al. (20) reported no significant changes in MVIC strength of the knee extensors and badminton-specific running and movement velocity after a 45 min simulated badminton match, although some increases in plasma CK activity and myoglobin concentration were observed. A simulated single badminton match played by state-level male players decreased muscle strength, voluntary activation, and muscle soreness of the knee extensors and flexors at immediately after and 1 h after the match, but they returned to the baseline by 1 day after the match (26). A simulated 3 h tennis match played on a hard court induced small increases in muscle soreness at 1–2 days post-match, and decreased muscle function assessed by one repetition maximum (1-RM) squat strength (35%), squat jump (7%), and counter movement jump (10%) at immediately post-match, and at 1 day post-match to a smaller extent (17). However, it is not known the level of muscle damage experienced by elite players who played longer singles matches in the grand slam tennis events in which some matches exceed 3 h.

It is likely that muscle damage carries over in the next match when a time to recover from a previous match was not long enough for some team sports such as soccer and rugby, and racket sports such as tennis. However, it is not known how these are considered for match intervals during official tournaments such as World Cups and Olympic Games. It is important to clarify the recovery time after a match, and examine a match interval in sports, starting from some major events. Therefore, we analyzed the interval between matches of men and women in team sports and racket sports (singles) in major sporting events held in 2021–2023, and reviewed literatures to find information about muscle damage and recovery in these sports to examine whether a recovery time required from a previous match are considered in a match schedule.

## 2 Methods

The study was approved by the Research Ethics Committee of National Taiwan Normal University (Approval #: 202311HS022). The procedures used in the study adhered to the tenets of the Declaration of Helsinki.

### 2.1 Interval between matches

We included soccer, rugby, field hockey, basketball, handball, volleyball, baseball, cricket, softball, badminton and tennis in this study. We focused on top-level competitions of these sports and examined the events held in 2022 and 2023 for the World Cups, 2020 Tokyo Olympic Games held in 2021, and singles in four major tournaments in tennis (Australia Open, French Open, Wimbledon and US Open) held in 2023. The 2023 World Baseball Classic was used for baseball, and the 2023 World Cup for cricket. The sporting events included in the present study are shown in Table 1. We obtained the information about intervals between matches from the websites of the above events held in 2022 and 2023 (e.g., 2023 FIFA women world cup: https://en.wikipedia.org/wiki/2023_FIFA_Women%27s_World_Cup), and 2020 Tokyo Olympic Games (e.g., https://en.wikipedia.org/wiki/Football_at_the_2020_Summer_Olympics). We analyzed the intervals (days) between matches of men and women in these sporting events, identified the ranges, and calculated mean ± SD interval for each sport event.

**Table 1:** The sporting events analyzed for intervals between matches in the present study.

Sport	World cup		Olympic	
	Men	Women	Men	Women
Soccer	2022 FIFA Men's World Cup	2023 FIFA Women's World Cup	2020 Tokyo	2020 Tokyo
Rugby	Rugby World Cup 2023 (Rugby 15)	Women's Rugby World Cup 2022	—	—
Field Hockey	2023 Men's FIH Indoor Hockey World Cup	2023 Women's FIH Indoor Hockey World Cup	2020 Tokyo	2020 Tokyo
Basketball	2023 FIBA Basketball Men's World Cup	2022 FIBA Women's Basketball World Cup	2020 Tokyo	2020 Tokyo
Handball	2023 IHF Men's World Championship	2023 IHF Women's World Championship	2020 Tokyo	2020 Tokyo
Volleyball	2022 FIVA Volleyball Men's World Cup	2023 FIVA Volleyball Women's World Cup	2020 Tokyo	2020 Tokyo
Badminton -Singles	2023 BWF World Championships	2023 BWF World Championships	2020 Tokyo	2020 Tokyo
Tennis -Singles	—	—	2020 Tokyo	2020 Tokyo
	2023 Australian Open	2023 Australian Open	—	—
	2023 French Open	2023 French Open	—	—
	2023 Wimbledon	2023 Wimbledon	—	—
	2023 US Open	2023 US Open	—	—
Baseball	2023 World Baseball Classic	—	2020 Tokyo	—
Cricket	2023 ICC Men's Cricket World Cup	2023 ICC Women's Cricket World Cup	—	—
Softball	2022 WBSC Men's Softball World Championship	2021 WBSC Women's Softball World Cup	—	2020 Tokyo

BWF, badminton world federation; FIBA, fédération international de basket-ball; FIFA, fédération internationale de football association; FIH, international hockey federation; FIVA, federation internationale de volleyball; ICC, international cricket council; IHF, international handball federation; US, United States; WBSC, world baseball softball confederation.

### 2.2 Literature review

We conducted a narrative literature review to obtain information about muscle damage and recovery relating to soccer/football, rugby, field hockey, basketball, handball, volleyball, badminton, tennis, baseball, cricket, and softball. The literature search focused on peer-reviewed journal articles published up until June 2024 using three databases (PubMed, Scopus, Google Scholar). Search keywords included: “soccer/football,” “rugby,” “field hockey,” “basketball,” “handball,” “volleyball,” “badminton,” “tennis,” “baseball,” “cricket,” “softball,” “match,” “game,” AND “muscle function,” “muscle dysfunction,” “muscle damage,” “muscle injury,” “MVIC strength,” “muscle strength,” “strength,” “isometric strength,” “delayed onset muscle soreness,” “muscle soreness,” “muscle pain,” “range of motion,” “countermovement jump,” “vertical jump,” AND “recovery”. We searched articles written in English related to ‘muscle damage’ and/or ‘recovery’ for the sports included in the analyses of match-intervals (i.e., soccer/football, rugby, field hockey, basketball, handball, volleyball, badminton, tennis, baseball, cricket, softball). We focused on changes in muscle functions [e.g., MVIC strength, range of motion, countermovement jump (CMJ)], delayed onset muscle soreness (DOMS)/muscle soreness] and their time course of recovery following a single match due to these variables are the main indirect markers of eccentric exercise-induced muscle damage used in previous studies (2, 6, 7). If studies only measured dependent variables (i.e., muscle function, performance) at one time-point post-match, they were excluded since no information of recovery from a match could be obtained.

### 2.3 Statistical analyses

The descriptive data are presented as ranges and their mean ± SD for each sport event. All statistical analyses were performed using the Microsoft Excel Version 2023.

## 3 Results

### 3.1 Match interval

Table 2 shows the intervals (days) between matches (range, mean ± SD) in the sporting events for each sport. The match-intervals differ largely among sports ranging from 0 day to 17 days. The longest match-interval was found for rugby (5–17 days), and the shortest interval was seen for softball (0–2 days).

**Table 2:** The rest interval (days) between matches (range, mean ± SD) in sports included in the world cups (2022–2023) and Tokyo Olympic games (2021), and the four grand slam events in tennis (Australia open, French open, wimbledon and US open).

Sport	World cup		Olympic	
	Men	Women	Men	Women
Soccer	2–7	3–7	3–4	3–4
	4.1 ± 0.7	4.9 ± 0.8	3.0 ± 0.2	3.1 ± 0.2
Rugby	5–15	6–8	5–17	6–17
	8.2 ± 1.6	6.9 ± 0.6	10.7 ± 4.7	10.7 ± 4.7
Field Hockey	2–7	1–7	1–2	1–2
	3.1 ± 1.2	2.6 ± 1.3	1.6 ± 0.5	1.7 ± 0.5
Basketball	1–3	1–2	2–3	2–3
	2.0 ± 0.4	1.3 ± 0.5	2.7 ± 0.5	2.7 ± 0.5
Handball	2–3	2–3	2–2	2–2
	2.0 ± 0.1	2.1 ± 0.0	2.0 ± 0.0	2.0 ± 0.0
Volleyball	1–3	1–3	2–2	2–2
	1.4 ± 0.6	1.5 ± 0.6	2.0 ± 0.0	2.0 ± 0.0
Badminton	1–2	1–2	1–4	1–4
	1.4 ± 0.5	1.2 ± 0.4	2.0 ± 1.0	1.8 ± 0.9
Tennis	—	—	1–3	1–2
			1.7 ± 0.6	1.3 ± 0.5
Australia Open	1–3	0.5–4	—	—
	1.9 ± 0.3	1.9 ± 0.7		
French Open	1–4	1–5	—	—
	2.3 ± 0.6	2.3 ± 0.5		
Wimbledon	1–3	1–4	—	—
	1.8 ± 0.8	2.2 ± 1.0		
US Open	1–3	1–6	—	—
	2.2 ± 0.5	2.1 ± 0.6		
Baseball	1–3	—	1–4	—
	1.5 ± 0.9		1.7 ± 0.7	
Cricket	3–9	1–5	—	—
	4.5 ± 1.4	2.8 ± 1.0		
Softball	1–1	0–1	—	1–2
	1.0 ± 0.0	0.9 ± 0.3		1.2 ± 0.4

### 3.2 Muscle damage and recovery

Table 3 shows changes in muscle functions and/or performance measures after a match or a simulated match of each sport. In many of the studies, a full time-course of the recovery was not investigated (e.g., 10–14, 16, 17, 19, 21–23, 25, 26), thus the time for the measures to return to the baseline was not clear for many sports. No previous study has investigated changes in muscle function and/or performance measures after a match of volleyball, softball and cricket (Table 3). Based on the available information, the recovery is shorter for badminton, when compared with soccer and rugby, and the longest recovery time was found for rugby (Table 3).

**Table 3:** The information of gender, age, performance level, duration of a match, and muscle function and/or performance variables, and delayed onset muscle soreness (DOMS) after a match of each sport in the studies. The last time taken the measurements (time of the last measure) and when the measurements were returned to the baseline (time of recovery) are shown.

Sport	Study	Sex age (y)	Performance level	Duration (min)	Variables	Time of the last measure (h)	Time of recovery (h)
Soccer match	Tanabe et al. (27)	Male 20.0 ± 1.0	15 university soccer players	90	CMJ DOMS RJ-index	48 48 48	0.5 Not full recovery 48
Soccer match	Junior et al. (28)	Female 23.6 ± 5.4	10 Brazilian professional soccer players	90	CMJ 10 m sprint 20 m sprint DOMS	48 48 48 48	48 24 24 Not full recovery
Soccer match	Dewangga et al. (29)	Female ≤ 17.0	20 U17 soccer players	90	Vertical jump	48	24
Friendly match	Marqués-Jiménez et al. (30)	Male 22.9 ± 2.4	10 semi-professional soccer players		RSA	48	24
Soccer (LIST)	Chou et al. (3)	Female 21.0 ± 1.1	12 elite university soccer players	90	MVIC-KE	120	120
					MVIC-KF	120	120
					CMJ	120	120
					30 m sprint	120	72
					Balance	120	120
					Agility *T*-test	120	120
					6 × 10 m	120	120
					YYIR1	120	96
					DOMS-KE	120	120
					DOMS-KF	120	120
Soccer (LIST)	Thomas et al. (23)	Male 21.0 ± 1.0	15 semi-professional players	90	MVIC-KE	72	Not full recovery
					CMJ	72	Not full recovery
					RSI	72	72
					Broad jump	72	48
					DOMS-KE	72	72
Soccer (LIST, match)	Magalhães et al. (12)	Male 21.3 ± 1.1	16 soccer players from 2nd and 3rd Portuguese divisions	90	MVIC-KE	72	Not full recovery
					MVIC-KF	72	Not full recovery
					CMJ	72	Not full recovery
					20 m sprint	72	Not full recovery
					DOMS	72	Not full recovery
Soccer (match)	Ispirlidis et al. (10)	Male 20.2 ± 0.8	12 elite soccer players	90	1RM	144	96
					20 m Sprint	144	120
					CMJ	144	72
					ROM-KE	144	96
					DOMS	144	96
Soccer (friendly match)	Brownstein et al. (22)	Male 21.0 ± 1.0	16 semi-professional players	90	MVIC-KE	72	72
					CMJ	72	48
Rugby (professional rugby league match)	McLellan et al. (13)	Male 24.3 ± 3.6	12 professional rugby league players	80	DOMS-KE ftCMJ rpCMJ DOMS	72 144 144 144	72 144 Not full recovery 96
Rugby (professional rugby league match)	McLellan et al. (14)	Male 19.0 ± 1.3	17 elite Rugby League players	80	pfCMJ	120	24
					RFD-CMJ	120	48
					ppCMJ	120	48
Rugby (match)	Roe et al. (21)	Male 17.4 ± 0.8	14 academy rugby union players	80	CMJ	72	72
					Plyometric push-up	72	48
Rugby (rugby union match)	Silva et al. (31)	Male 28.9 ± 3.5	14 amateur rugby players	80	10 m sprint	96	72
					L-shape run test	96	Not full recovery
Rugby (RLMSP)	Twist and Sykes (15)	Male 23.5 ± 2.3	10 university rugby players	86	MVIC-KE	48	48
					MVIC-KF	48	Not full recovery
					CMJ	48	48
					DOMS	48	Not full recovery
Field hockey (match)	Burt et al. (24)	Male 20.0 ± 2.4	11 field hockey players	70	MVIC-KE	48	No change
					MVIC-KF	48	No change
					CMJ	48	No change
					DOMS	48	48
Basketball (match)	Moreira et al. (18)	Female 27.4 ± 4.8	11 elite professional basketball players	40	1RM leg press	24	24
					1RM bench press	24	24
					Agility *T*-test	24	24
					DOMS-KF	48	Not full recovery
Basketball (simulated match)	Pliauga et al. (19)	Male 21.5 ± 1.7	10 first in the Lithuanian National Basketball division players	40	CMJ	48	Not full recovery
					10 m sprint	48	Not full recovery
Volleyball	No study						
Handball (match)	Chatzinikolaou et al. (16)	Male 22.8 ± 1.4	24 elite adult team handball players	60	CMJ	144	48
					1RM-leg press	144	48
					1RM-bench press	144	48
					Handgrip	144	48
					10-m sprint	144	48
					Line drill test	144	48
					Agility *T*-test	144	48
					DOMS-KE	144	48
					DOMS-KF	144	72
					dROM-KJ	144	72
					ndROM-KJ	144	No change
Baseball-pitching (simulated pitching)	Reinold et al. (11)	Male 26.0 ± 4.9	67 professional baseball pitchers	50–60 pitches at full intensity	ROM-shoulder ER	48	No change
					ROM- shoulder IR	48	Not full recovery
					ROM- elbow flexion	48	No change
					ROM- elbow extension	48	Not full recovery
Softball	No study						
Badminton-singles (simulated match)	Lin et al. (26)	Male 26.4 ± 5.3	10 elite singles badminton players from the Western Australia badminton team	60	dMVIC-KE	24	24
					ndMVIC-KE	24	24
					dMVIC-KF	24	24
					ndMVIC-KF	24	No change
					DOMS	24	Not full recovery
Cricket	No study						
Tennis-singles (simulated 3 h tennis match)	Gomes et al. (17)	Male 17.6 ± 1.4	10 young tennis players from Brazil national junior ranking 10th–45th	180	1RM	48	24
					CMJ	48	24
					SJ	48	24
					DOMS	48	Not full recovery

LIST, Loughborough intermittent shuttle test; RLMSP, rugby league match simulation protocol (RLMSP is one of simulated rugby matches); CMJ, countermovement jump; d, dominant limb; DOMS, delayed onset muscle soreness; RSA, repeated sprint ability; ER, external rotation; nd, nondominant limb; ftCMJ, flight time of CMJ; IR, internal rotation; KE, knee extensors; KF, knee flexors; PF, plantar flexors; MVIC, maximal voluntary isometric contraction strength; pfCMJ, peak force of CMJ; ppCMJ, peak power of CMJ; 1-RM, one repetition maximum; RFD, rate of force development; RFD-CMJ, rate of force development during countermovement jump test; RJ-index, rebound jump index; ROM, range of motion; ROM-KJ, knee joint of ROM; rpCMJ, relative power of CMJ; RSI, reactive strength index; SJ, squat jump; YYIR1, yo-yo intermittent recovery running level 1.

## 4 Discussion

The match intervals varied among sports and events such that the intervals in World Cups and Olympic Games were the shortest for softball (0–2 days) and the longest (5–17 days) for rugby (Table 2). Soccer matches were generally scheduled with 2–7 days of rest between matches in the World Cups, but 3–4 days during the Olympic Games, and rugby had a longer interval for men (5–15 days) than women (6–8 days) in the World Cups (Table 2). It appears that the recovery time is somewhat considered in scheduling matches in the events, since the recovery takes longer after rugby or soccer matches than softball matches (Table 3). However, considering the wide range of intervals between matches, and possible large intra- and inter-individual variability in external and internal load in a competition, some athletes are unlikely to recover from a previous match, but still play a following match (e.g., 31).

As shown in Table 3, muscle function and/or performance parameters were impaired following a match for all sports, but the time for them to return to the baseline differed largely among sports. No information about changes in muscle function and/or performance after a match was found in published papers for volleyball, softball and cricket. The extent of decrement in muscle function that elite athletes experience after an official match is not necessarily clear, since many of the studies did not use them as participants (3, 10–12, 15–19, 21–27, 29, 30). Thus, in order to obtain the whole pictures of recovery after matches for elite athletes, more studies are required.

It is well-documented that muscle damage represented by DOMS and prolonged decreases in muscle function is induced by unaccustomed exercises consisting of large volume and/or high-intensity eccentric contractions, and the magnitude of muscle damage is reduced when the same exercises are repeated, known as the repeated bout effect (2, 32–34). In spite of the repeated bout effect, some muscle damage is still induced in well-trained athletes (3, 5, 8, 10, 13, 18, 27, 29, 30). The muscle damage impairs performance, which could last for several days (6–8, 35). It is important to note that muscle damage is induced to some extent even for well-trained athletes who are accustomed to most of the eccentric-biased movements performed in sports (5, 8, 10, 13, 18). It is likely that eccentric-biased movements performed in sports such as rapid deceleration, sudden stop, fast change of direction, landing, jumping, hopping, cutting, body collision/contact and preventing falls are the main causes of the decreases in muscle function and performance parameters (7, 36). It is likely that more strenuous eccentric-biased movements are required in a competitive match than in training and practice sessions, which could exceed the level of the repeated bout effect.

Fédération Internationale de Football Association (FIFA) sets each team must have at least 48 h of rest before the next match played in its official tournaments (https://digitalhub.fifa.com/m/2744a0a5e3ded185/original/FIFA-World-Cup-Qatar-2022-Regulations_EN.pdf). However, it seems possible that players required more than 48 h to recover from soccer matches even for well-trained players. Koyama et al. (37) showed that the relative external load of players increased when the competitive level of the opponents increased, suggesting that internal and external loads varied depending on contextual factors. It has been reported that external load and internal load differ between World Cup matches and friendly matches (38). Silva et al. (31) compared the impact of simulation matches and real matches in their systematic review article, and stated that the real matches (11 vs. 11 format) induced greater magnitude of DOMS and increased CK activity in the blood, although neuromuscular alterations were similar. Chou et al. (3) and Tseng et al. (9) reported that it took 4–5 days for elite female soccer players to restore MVIC strength of the knee extensors and flexors and performance measures such as Yo-Yo intermittent recovery test level 1 to the baseline levels following a 90 min LIST (3, 9). Thus, it seems likely that actual soccer matches could induce greater muscle damage than the LIST, because of other activities with a ball, and competitive nature of matches including contacts and impacts with opponents, thus the recovery takes longer. However, some studies reported that muscle soreness and some performance measures (sprint, vertical jump) fully returned to baseline at 24–48 h following a 90 min soccer match (27, 28, 30). It appears that many factors such as physical and mental demands in matches, environment and condition (e.g., temperature, humidity, ground condition) and the level of players affect the magnitude of muscle damage.

Based on the data from the female soccer players in the study by Chou et al. (3), and possible greater physical and physiological demands in competitive matches in major events such as World Cup, it is assumed that players need to have more than 4 days between matches. However, the interval between matches was 3–7 days for the group rounds, and that for the quarter finals, semifinals, third place and final was 3–6 days in the 2023 FIFA Women's World Cup. It is possible that a team that had a shorter interval between matches has disadvantage against a team that has a longer interval between matches. It is interesting to note that the interval between matches was not the same in 6 out of 8 matches in the quarter-final, semi-final, final and the third-place matches between teams, and 5 out of 6 matches (83%) were won by the teams who had a longer interval from the previous match in the 2023 FIFA Women's World Cup (8).

Although it is ideal to have the same match interval for the teams competing each other from the point of fairness, this would be almost impossible from the organizational perspective. Thus, it is important to find strategies to facilitate recovery after a match to prepare players for subsequent competition during sport events. One of the strategies is to use therapeutic modalities such as far-infrared ray (FIR) lamp therapy that has been shown to enhance recovery from muscle damage and performance parameters following a single bout (9) or multiple bouts of LIST (39).

It has been reported that basketball players in the National Basketball Association (NBA) teams scored more with a 2-day rest interval between matches when compared to consecutive matches (40), and had less successful three-point shots per 100 possessions and 20% less dunks during the fourth quarter compared to the first quarter when matches were played on consecutive days (41). The interval between matches in rugby (7–9 days) is longer than that in soccer (2–7 days) as shown in Table 2. The full recovery of muscle function and performance from a soccer (3–5 days) and rugby (2–6 days) match seems similar (Table 3). The playing time is shorter for rugby (80 min) than soccer (90–120 min); however, the total numbers of contact conditions and the extent of the contact impacts are likely to be greater for a rugby match (e.g., tackling: 156 times/match, scrums: 22 times/match, rucks: 16 times/match; total: 294 times/match) (42) than soccer (no tackling is allowed; ∼147 times body collision/match) (43). This may be a reason for a longer interval between matches in rugby than soccer. The full recovery of muscle function and performance after a field hockey match took about 1–48 h, while that following badminton and tennis singles matches took less than 2 days (Table 3). It is likely that the magnitude and volume of eccentric-biased movements are less for badminton and tennis than those in soccer and rugby. However, when a match duration is long such as more than 3 h in tennis for example, it is likely that a large amount of eccentric-biased movements contractions of lower limb muscles are performed, resulting in muscle damage and impaired performance. It is interesting to investigate how tennis players recover from a previous long match and are ready for the next match within 2 days. It is also possible that the interindividual responses differ in different conditions due to external-internal load influenced by match duration and contextual factors such as opponents’ level and fixture congestion (44).

When players perform matches without full recovery from previous matches, it may result in accumulative physical and mental fatigue, compromise performance and increase injury risks of players (40, 45, 46). In fact, previous studies reported that a match congestion increased non-contact injuries in professional soccer players (45, 47–50), field hockey players (51) and basketball players (52). For example, Dupont et al. (45) reported that professional male soccer players played two matches per week without affecting the distance covered and the numbers of sprint during matches, but it increased the injury rate 6 folds when compared with a match per week. Mason et al. (51) showed that field hockey matches with 24 h interval had 3.8–6.8 times higher injury risks than matches with 3–7 days interval. Additionally, athletes have more risks of illness in congested match conditions (53–56). For example, Schwellnus et al. (55) stated that a congested match schedule increased risk of both subclinical immunological changes that could increase the risk of illness, and actual symptoms of illness or diagnosed illness. In the study of English Premier League, Morgans et al. (53) showed that playing 5 matches in 15 days led to large decreases in salivary immunoglobulin-A (SIgA) concentration (e.g., 2-day post-match 3: −68%, 2-day post-match 5: −71%). Therefore, it is important to examine adequate intervals between matches to minimize injury risks and illness of the players. Sport organizations and/or sports event organizers should consider the time taken for players to recover from a previous match.

It is also interesting to examine if a shorter interval between matches lowers the match quality, and higher quality matches can be seen when athletes are competing with enough recovery from previous matches. In fact, Folgado et al. (57) examined the physical and tactical performances of an English professional football team under congested (played one match every 3 days interval for 3 matches) and non-congested (played one match at least 6 days rest for 3 matches) fixture periods. They reported that no differences in the physical performances such as the total distance covered between congested and non-congested matches, but players spent more time for movement synchronized during the non-congested fixtures (lateral displacements: 41.3%, longitudinal displacements: 77.2%) when compared with congested fixtures (38.5%, 74.5%). The authors stated that the reduction of synchronization could be associated with an increased perception of fatigue (57).

It may be difficult to change match-intervals in sporting events, since it affects the budget and schedule of players in a season for other events. Pillay et al. (58) conducted a survey study of 1,055 professional male soccer players around the world, and found that 76% of them thought that there should be regulations in place to protect them from insufficient breaks (i.e., not enough rest in both off-season and in-season). Coaches and medical practitioners need to have strategies to reduce fatigue and muscle damage in matches, as well as facilitate the recovery of athletes after matches. It is necessary to have a simple measure to assess status of athletes including their readiness for the next match. In soccer, we reported that counter movement jump height could indicate recovery from a previous match well (3). Sporting organizations should communicate with coaches and athletes to set a schedule of events, and they should also seek medical and scientific guidance before making a match calendar (53). An adequate interval between matches should be discussed more openly for each sport. To minimize injuries and accumulative fatigue as well as prevent illness in sporting events, effective recovery strategies are important to be developed and established, warranting more studies. It is also important to monitor match intervals in upcoming major sporting events in 2024 such as the Olympic Games Paris and beyond.

There are several limitations in the present study. First, the present study included only 11 sports (soccer, rugby, field hockey, basketball, volleyball, baseball, cricket, handball, softball, badminton, tennis) among many, and focused on World Cups held in 2023, Olympic Games in 2021, and tennis gland slams in 2023. It is interesting to extend the analyses to previous events held before the ones included in the present study. Second, we searched articles relating to the 11 sports in which muscle damage and recovery were investigated using the literature review, but it was not based on a systematic review protocol. Thirdly, the present study included studies with participants who were not necessarily elite athletes. Since limited studies used elite athletes, and it is possible that they could recover from a match in a shorter time, actual recovery profiles of elite athletes who represent countries in the major sporting events are largely unknown. In order to better understand muscle damage and recovery in sporting events, more studies are required using top levels athletes, and barriers to conduct such studies requires good collaboration with teams, athletes and sporting organizations. Fourthly, LIST may not represent contemporary soccer matches and may demands of a female match-play. Magalhes et al. (12) reported that changes in muscle damage and performance parameters following a 90 min LIST played by male players of the second and third Portuguese divisions were similar to those after a 90 min soccer match. However, the LIST does not include soccer specific skills such as passing, kicking and heading a ball. Future studies are warranted to examine the effects of congested matches on muscle damage, fatigue, performance and health.

In conclusion, the present study showed that the interval between matches varied among sports and events, and the match intervals in the major sporting events were not necessarily long enough for the players to fully recover from the previous match in many cases. It may not be that sport organizations and event organizers can arrange the rest interval based on the full recovery time. More studies are warranted to investigate what the best rest interval is to be set between matches for each sport event such as World Cup and Olympic Games. Since the recovery time is crucial, it is necessary to for an event organiser to schedule matches to give the same match interval to the teams competing against at least.

## Data Availability

The datasets are presented in the article, further inquiries can be directed to the corresponding author.
